# Supplementary material for: Mobile genetic element-encoded putative DNA primases composed of A-family polymerase—SSB pairs
Source: Front Mol Biosci. 2023 Mar 16;10:1113960. doi: 10.3389/fmolb.2023.1113960 (PMC10061031; doi:10.3389/fmolb.2023.1113960)
Supplement: Supplementary file 1 [file DataSheet4.DOCX]

Figure S4: Alignment of all “large” Pol proteins with the 1qsl.pdb - a structure of the Klenow fragment of *E. coli* DNA Pol I, which lacks the 5’-3’ exonuclase but includes the 3’-5’ exonuclease and the polymerase domains.

Promals3D was used to create this alignment.

Active site residues are marked with asterisks over the sequences (blue for the 3’-5’ exonuclease and red for the polymerase sites), and Helix O of the *E. coli* Pol I, which makes important interactions with substrates, is also labeled. The helix that is shorter in the predicted structures, relevant to binding of the 1^st^ dNTP, is marked with #.

Active site:

1qsl 324 -------------------------------------MISYDNYVTILDEETLKAWIAK--------LEK 348

T_sanguinis 1 ---MKEFFELQN-DFDFKLEGDKLIPT-FKNQ-NQQTLTSGQVDNFTTVKKYSTSVKAYLNNPP---QEY 61

S_qingdaonense 1 MISMDALAVLQD-SFDISLDGNDLVLH---PKAALPEDRSTMYEAMVQIKQHKANAVYRLNAPS---NAY 63

M_jejuensis 1 -----MLQLAQK-YFEISLLDQKVRCQ---PKSEDVLTNQQAIKLLTDLRRHKQNTMFLLSNQGQSLLTA 61

P_pinistramenti 1 ---MKTLKMLKSLGYKLEPSGSGILYE-SPSGIAP--DPALVAACLNDIRRNKALVRELLTRPI---PEY 61

N_nealsonii 1 ---MKELRELQKAGYKFIIDGGNVSYQNPNRLNPL--NADSFRNKLVHLKQQKHRVYKFLLKQK---VPP 62

C_difficile 1 ---MNILRELEELGYKLKVREGNIEFE---YRGKGVNDYEKSLELLYEVKKRKIRATNYLLCNY---KQY 61

C_perfringens 1 -------------------------------------------------------------------MNK 3

B_weidmannii 1 -------------------------------------MVVRSIV-----GNFNDKTLIL--------NEK 20

T_pasteurii 1 ------------------MTH-------------------------------------FQQHHD--VGVG 13

L_newyorkensis ----------------------------------------------------------------------

S_aureus_V ----------------------------------------------------------------------

S_aureus_mer ----------------------------------------------------------------------

[Consensus_aa:](http://prodata.swmed.edu/promals3d/info/consensus.html) ......................................................................

[Consensus_ss:](http://prodata.swmed.edu/promals3d/info/consensus_ss.html) hhh hhhhhhh h

Active site: *** ***

1qsl 349 APVFAFDTETDSLDNISANLVGLSFAIEPGVAAYIPVAHDYLDAPDQISRERALELLKPLLEDEKALKVG 418

T_sanguinis 62 SKIVGVDIETTGLDCFKDRIRLIAVYG-DDFSYVGDK----------------LQEVEDILSDSTVLKVF 114

S_qingdaonense 64 ANIVAFDLETSDLDPLKGEIKLISVWG-DGVEKVTSD----------------VYEVEELLRDPSILKVC 116

M_jejuensis 62 TTLVAVDIETTSLNPSDGDIRLISVFS-EENQFVTED----------------VAQVADILKDESILKVF 114

P_pinistramenti 62 RNVLAVDIEVIG-----DTIRLISWAD-GESSGASEN----------------LSLLADKLKDPAVLKVF 109

N_nealsonii 63 KAVVAVDIETYG-HYTDGMIRLISWAD-QNGCYVSES----------------IDDIKELLENPEVLKVF 114

C_difficile 62 KKIIAIDIETTATMPQEGKIVLIAMHD-NEGGKVAYD----------------PKEIEDILADEDILKVF 114

C_perfringens 4 LKVVFIDIETSSLYFMKGVIRLVAFSDGSSKLLTRTV---------------VDDDLRKILKNEKILKVF 58

B_weidmannii 21 EIN-----TIRLKEVLVNSDVLKVVPD----VSLVCNLAS--------KGVQIT----SWIT---ITTL- 65

T_pasteurii 14 RYGL---------------------------------------------------------------ADI 20

L_newyorkensis 1 --------------------------------------------------------------------MP 2

S_aureus_V 1 ---------------------------------------------------------------------M 1

S_aureus_mer ----------------------------------------------------------------------

[Consensus_aa:](http://prodata.swmed.edu/promals3d/info/consensus.html) ...................................................................p*h*.

[Consensus_ss:](http://prodata.swmed.edu/promals3d/info/consensus_ss.html) eeeeee eeeeeee ee hhhhhhhh eeee

Active site: *****

1qsl 419 QNLKYDRGILANYGIELRGIAFDTMLESYILNSV------AGRH-DMD---SLAERWLKHKTITFEEIAG 478

T_sanguinis 115 HNALFDVSFLKVAGIEVKNY-SDTIVMAKIISNS-----AVFD--SLE---YLAKKYLNLELDK--ELQH 171

S_qingdaonense 117 HNASFDVTFLRTKGITITSY-MDTLVMAQVFHNR-----VKTDN-SLK---GLAEKYLGRNLNK--NLQH 174

M_jejuensis 115 HNAAFDATWLTAKGYPVVNY-TDTMLMGQVLHNT-----AKSNN-SLL---ALAFEHLGIILDK--TLQA 172

P_pinistramenti110 HNALFDVPLLLKNGFIVSNY-TDTMVLAQVLHNR-----VAQEN-GLA---DLAWKYLGIQLDK--TLQA 167

N_nealsonii 115 HNALFDVTWLLAKGINVQTY-TDTKVLSQIINNR-----ISKDN-SLE---ILSLKHFGILLDK--SLQS 172

C_difficile 115 HNASFDVVWFEYFGYKVNEY-TDTMIMSQVINNR-----VRQEN-SLV---DVAHKYLNVVMSK--QLQD 172

C_perfringens 59 HNAKFDVEFFIHNGYEVNNY-SCTLVMAQVLGE--------KEL-SLK---ALCNKYLGVVIDK--SMQH 113

B_weidmannii 66 -------------------KMLAC--DDSIYTS------------------------------------- 77

T_pasteurii 21 DLPY--------------------SSLQKILLLP------LNY--------DVE---------------- 40

L_newyorkensis 3 EQRM--------------------FDLKRLLLLP------DTI--------DME---------------- 22

S_aureus_V 2 YQ---------------------ANIRDLITKLPQSNKTEHFLMNKFSNQDKVQQLQRQ----------- 39

S_aureus_mer 1 -----------------------METRDKLMSLTQSDKTQQWLMDKSSNQDDIQQLQQQ----------- 36

[Consensus_aa:](http://prodata.swmed.edu/promals3d/info/consensus.html) pp.....................*h*b..sp*lh*..............p*h*.....*l*.bbbb............

[Consensus_ss:](http://prodata.swmed.edu/promals3d/info/consensus_ss.html) ee hhhhhhhhh hhhhhhhhh hh hhhhhhh

Active site: *** ***

1qsl 479 KGKNQLTFN-QIALEEAGRYAAEDADVTLQLHLKMWPDLQKH-----KGPLNVFENIEMPLVPVLSRIER 542

T_sanguinis 172 ----SGNWQ-AELTQEHHDYCLKDAEVTLKLFRVLYDLIVERY------LFPTY-RTEISALPSLIELQT 229

S_qingdaonense 175 ----ADHWQ-GDLTEDHYAYALLDAQVALELYYFLQEQITHLH------LDVVL-KREMAAIDTIIELNR 232

M_jejuensis 173 ----ETNWQ-SDITEDHKRYALKDAEVTYHLYHQLKAKIAEKH------LDVVL-DREVAMLPVVVLLNL 230

P_pinistramenti168 ----EAHWE-GELTEAHYAYSRRDAEVTYQLYHILTDRLEDRY------LEGVA-RREIAALPAAVHLQM 225

N_nealsonii 173 ----ENNWN-GKITNEHKEYAKRDAEVTYKLYFRLLQIIEESS------LEEVM-LREISALPAVIEMQL 230

C_difficile 173 ----TSNWNNGVITKEHEEYCKRDAEITYKLYFTLMDEIHRLE------IYPVL-DREIRALPSIIELRR 231

C_perfringens 114 ----SENWNVEKLTQEHIDYVIQDVINTRLLYYKLLDELIKKN------LLETY-ERERKALTSVIMLEL 172

B_weidmannii 78 ----------------------NPTNLMI-TQWMER-VYRNS-------SKAAL-EREINLGPTLIELTL 115

T_pasteurii 41 -----------------------------SRGPHVRRILQASN-----VFSSLY-DLEKSIQPLLSTIES 75

L_newyorkensis 23 -----------------------------TSKSRTDKIIRTSR-----VFSELY-LLERQLNPILLGMQE 57

S_aureus_V 40 ------------------------------ISQQLDQQYNELLANEKAKLDQYV-EVHHNLEPLKKEIES 78

S_aureus_mer 37 ------------------------------FSQQLDQQYNALLADEKAKLDQYV-EVHQGLESLKEEIES 75

[Consensus_aa:](http://prodata.swmed.edu/promals3d/info/consensus.html) .............................p*h*...*h*.p.*h*pp........*h*..*hh*.p.**E**..*h*.s.*h*..*l*p.

[Consensus_ss:](http://prodata.swmed.edu/promals3d/info/consensus_ss.html) hhhhhhhhhhhhhhhhhhhhhhhhhhh hhhhh hhhhhhhhhhhhhhh

Active site:

1qsl 543 NGVKIDPKVLHNHSEELTLRLAELEKKAHEIAGE-EFNLSSTKQLQTILFEKQGIKPLKKTPSTSEEVLE 611

T_sanguinis 230 NGMMLDKSELRAYLEKLRVQHQELKERLEIQLAC--ENLNSPPQLLKSLQNLGVPIDNVEE-----KTLK 292

S_qingdaonense 233 NGIGFDYEGWEQELEYMNTESLQFQETVRNLLETPTLNLQSPAQLMAALGEQGIHVEGTSD-----EVLA 297

M_jejuensis 231 NGIPFDYAGWQQELDAIEKEASSLETYITELFQIPTLNLSSHQQVKAALLSEGIELDSVTD-----ESLA 295

P_pinistramenti226 AGIPFDFEGWKAELQHLEAEQLALQRTIELDLGCEGINLRSSQQLKEALLQLGIPVQGTAD-----EELA 290

N_nealsonii 231 YGMLFDYTGWKKELKKMKNEKNAIEEEIRSIFEHPTLNLQSPLQVKEALSTIGITVESTSE-----GALA 295

C_difficile 232 NGIKFEYGKWYMQILEYEREKDMIEKEIKSILNVKDLNLNSPIQVVEILNKFGINVTSSSD-----EELA 296

C_perfringens 173 NGIKMDFEKWDRVLDGDRQLCNEIEEEIKNILNLSELNLNSPKQLVESLYTYGIKLHSTSD-----DELA 237

B_weidmannii 116 NGIGFDFDNWMKELEDKKQKLSELKQEIQHLLDMPMLDVDDPFQLVKACKARAIILKSVTI-----DYLT 180

T_pasteurii 76 RGLIVAERWFSDVLPKRQDELNRTIEEISKYVLVEGNE-IDTSRVTDFLIRNDLP-AANDN-----DKFQ 138

L_newyorkensis 58 SGLLVSRTWLDEGLELTRQLLLTREAEIQAYIGKTAGL-IHAEKLATFLTEKKLP-EVRRT-----GDFK 120

S_aureus_V 79 ESINLDTDKLPDIKATMLE--------------------------------------------------- 97

S_aureus_mer 76 EPITLNIDKLPDIKATMLE--------------------------------------------------- 94

[Consensus_aa:](http://prodata.swmed.edu/promals3d/info/consensus.html) p**G***l*.*h*s.p.*h*.p.b...p.b...*h*ppp*l*p.*hh*.....p..ss.p*l*.p*h***L**.p..*l*...s.pp.....c.*h*.

[Consensus_ss:](http://prodata.swmed.edu/promals3d/info/consensus_ss.html) hhhhhhhhhhhhhhhhhhhhhhhhhh hhhhhhhhhh h hhhh

Active site: ##

1qsl 612 ELALDYPLPKVILEYRGLAKLKSTYTDKLPLMINPKT---GRVHTSYHQAVTATGRLSSTDPNLQNIPVR 678

T_sanguinis 293 KQSGMFPIVKELVQYKKLTKVLTTYGEKLLSLIETD----NRVRGNWNLIGTATSRMTCKGPNFQGIP-- 356

S_qingdaonense 298 KYEGQYDVIDSLRKYKKRRKQINAYGEKLKQAIGKD----GRLRGTWRLMGTDTFRMTCKQPNLQGMP-- 361

M_jejuensis 296 KVEHLHEVIPVLRKYKKLKKLLSTYGEKLAKHIGQD----GRLRGQWRVIGTDTSRMSCKAPNLQGLP-- 359

P_pinistramenti291 KHEAAHPVIPKLRRYKKIQKTLSAYGEKLHQQIGPD----GRIRGKWRLIGANTSRMTCNQPNLQGLP-- 354

N_nealsonii 296 SLEDQHQVIVLLRKYKKLQKRLSSFGEKLKERMGSE----GRIRGSWNVIGTNTGRMSCVKPNLQGLP-- 359

C_difficile 297 KFSDNNEVIKLIRKYKKLKKRISSFGDKLKEKIDND----GCIRGKWNLIGADTFRMTCTSPNLQGMP-- 360

C_perfringens 238 KYSESHEVIRLIRKYRRLKTKIRTYGEKMKTFICGD----GRIRGAWWLIGATSGRMSCNNPPLQAMP-- 301

B_weidmannii 181 HIKDEVPIARLLLKYQSIKKFIKQYGDNLGSHLSTL----NRIHGDWHGCGAFSGRMSCSKPNLQAFP-- 244

T_pasteurii 139 MYRNLHPLYELLLKNNKQQQFLKQWGNKLLAEGRRTES-GVVIKGNWQSFSSYSGRMFCKQLPLTSLP-- 205

L_newyorkensis 121 KNKAVHPIYPIFMQYQATKQFIDFWGTKLERESQKTSRGELQLKGQWTSFTSFSGRMFAKNLPLTSLP-- 188

S_aureus_V 98 ----------KAKNDEHFDKIEQL-FDRLDQSLNGT----NRLYTQLSLIGTRTHRITTKRFNVQGLP-- 150

S_aureus_mer 95 ----------RAKNDEHSDKIEKL-FDRLEQALNGT----NRLYTQLSLIGTRTHRITTKNFNLQGLP-- 147

[Consensus_aa:](http://prodata.swmed.edu/promals3d/info/consensus.html) c.p..*@*.*lh*..*h*.p.pp.pp.bp.*@*s**-+L**.p.*h*..p....sp*l***+**sp*@*p.*h*t*h*.o.**R***h*otpp.s**LQ**t*h***P**..

[Consensus_ss:](http://prodata.swmed.edu/promals3d/info/consensus_ss.html) hhhh hhhhhhhhhhhhhhhhhhhhhhhhh eeeeeee ee

Active site: #### *****

1qsl 679 NEEGRRIRQAFIA-PEDYVIVSADYSQIELRIMAHLSRDKGLLTAFAEGKDIHRATAAEVFGL---PLET 744

T_sanguinis 357 ----KEIKGFFHA-NPGHVFVIVDYSTVELRILAEISGCKKLIDAFNQGLDLHYETAKTVLGK-----EG 416

S_qingdaonense 362 ----GKAKPYFRP-EEGKSFIVADYRTIELRILAELSGDPELKQAFYDNEDLHTKTTAAILNK--QEGEM 424

M_jejuensis 360 ----SIAKPYVKA-SKGNVFVIADYSTIELRILAEITKDPELIAAFQTGEDLHAKTTGAVLGK--PPGQA 422

P_pinistramenti355 ----SIAKPYVKA-PAGRTLVIADYSQIELRVIAQLAGEERMIESFRSGEDLHYKTASLILGK---PVEE 416

N_nealsonii 360 ----SLAKTYVKA-SKDHLLLIADYSQIELRVMAQMAQDKVMMESFQNGVDLHLNTASEIMGK---PIIL 421

C_difficile 361 ----RVSKEYFVPRNKESVFVIADYSQIELRVLAEISNDKLLIDAFKNGEDLHRKTASMILDK---HISE 423

C_perfringens 302 ----GISREFFIA-EKGNKLVCADYSQVELRVLASISSDETLINYFKSGVDLHTGTASLVFKK---TIEE 363

B_weidmannii 245 ----SEAKKYFIP-KNDYVLISADYSQQELKMLAELSGCPRLRKAFTDGVDVHSLTASYLFNL---EVKN 306

T_pasteurii 206 ----KAVREFITS-PDERVILSLDFNNAELRILAYYSKCTKLLEQFEIGEDIHFQTGTMIAQA-IGLKDA 269

L_newyorkensis 189 ----RIMRPYVCS-GDDRCIYSLDFNQAELRFVAYYARCDALSLLINEQVDLHQQLGNIITSQIATTAQL 253

S_aureus_V 151 ----KLVQQMILP-SQFKKVYTIDFKSFEPSVAAYMTQDEQLIDYLNHEEGLYDALLRDL---------S 206

S_aureus_mer 148 ----KAVQHTILP-SKFKKVYTVDFKSFEPSVAAYMTQDSKLIDLLNQKDGLYDALLSEL---------G 203

[Consensus_aa:](http://prodata.swmed.edu/promals3d/info/consensus.html) ....p.*h***+**.*hh*.s..p.p.*hh*.*h***D***@*pp*h***EL+***lh***A**b*h*s.s..**L**bp.*h*pp..**D***l***H**..*hh*s.*lh*sb.....p.

[Consensus_ss:](http://prodata.swmed.edu/promals3d/info/consensus_ss.html) hhh eeee eeeeee hhhhhhhhhh hhhhhhhh hhhhhhhhh hhh

Active site: **helix O** *****

1qsl 745 VTSEQRRSAKAINF-GLIYGMSAFGLARQL------NIPRKEAQKYMDLYFERYPGVLEYMERTRAQAKE 807

T_sanguinis 417 ISDDERQIGKVINF-GLIYGLTAYGLMNEINQIPGFNINKEEAQNFIDMYFMNYKGVAKYKNQQLK---- 481

S_qingdaonense 425 VTAEERKIGKVVNF-GLVYGMTKWGLQKKIQGATGAAISLQEAETFRNRYFELYPGVLRYQDRMLQ---- 489

M_jejuensis 423 ITSEERKIGKVINF-GLLYGMTAYGLQRKIQAATGQVLSKDEAEVFRNRYFELYANVLTYQNQMLQ---- 487

P_pinistramenti417 VTPEERKIAKTTNF-GLLYGMQAYGLMKRIQTQCGIDISLGMATTFRNGYFYSYPAILRFQDYVLQ---- 481

N_nealsonii 422 VSSDERKIAKTTNF-GLLYGMTVYGLQKRINAAFGLDVSYETANLYRNGYFNLYKQVRSFQDAALK---- 486

C_difficile 424 VTDEERSIAKAANF-GLIYGMTSYGLQKKIKADYCMEITLEEAEKFRNGYFEAYKDVLKYQDKMLK---- 488

C_perfringens 364 ITKEERQVAKSLNF-GIVYGITSYGIQKNLRK-SGLNVSLEEAEEYRLEFLRVYPKIRELQDNLLR---- 427

B_weidmannii 307 ITSQQREIAKKVNC-SIIYGITGYGLAKYLSKALKQDVTLAEANQLKEKFFEAYPNIFQYQDALLT---- 371

T_pasteurii 270 NVAVIRKTGKQFAF-ALLYGAGTSMIVNNLRKLV-PTVTSATVSQLVTAFFKTYPEVHLFLDNLEK---- 333

L_newyorkensis 254 TDGQIRQMGKQFLF-SFLYGAGKNSLLQSLQKHL-PEMTSIGVERIVNTFYKRFPALQLSLRALEK---- 317

S_aureus_V 207 LSKEKRVSVKRAFIGSFLFGGRYSS-SKFKIN---QEVSEIN----WLQVMSKFKKVIEFKEQVEK---- 264

S_aureus_mer 204 LSDELRVFVKRAFIGSFLFGGNFKN-PKFKLN---QYVSEVQ----WLDAVSQFTKVIELKKHVEK---- 261

[Consensus_aa:](http://prodata.swmed.edu/promals3d/info/consensus.html) *l*ospb**R**p*h*t**K**.*h*.*h*.t*hl***YG***h*s..t**L**.p.*l*p..*h*...*l*o..p*h*pp*h*...*@h*p.*@*..*l*.p*h*bc.*h*bp....

[Consensus_ss:](http://prodata.swmed.edu/promals3d/info/consensus_ss.html) hhhhhhhhhhhh hhhh hhhhhhhhhhh hhhhhhhhhhhhhh hhhhhhhhhhhh

Active site:

1qsl 808 QGYVETLDGRRLYLPDIKSSNGARRAAAERAAINAPMQGTAADIIKRAMIAVDAWLQAEQPRVRMIMQVH 877

T_sanguinis 482 SPMVTTLGGRYWDSSNGLK------LLKDNQRLNYAIQASCAEGLKESLALLMMEK---KDTWRLVGAIH 542

S_qingdaonense 490 RSFIQTLGGRYWSDTHHML------EKGSIARYNYPIQATSTEGLKEALALFLEGR---QKTWKLAAVVH 550

M_jejuensis 488 SDCIFTLGGRYWSAETTEL------RKGAISRFNYPIQSSGAEGVKEALKFLLPKL---QEEWKLIAAVH 548

P_pinistramenti482 ADRLETLGGRYWCPGHAGL------KKGAIQRLNYPVQATAAEGFKEAQALLIEEL---PSDWLLIAAVH 542

N_nealsonii 487 ADLIETMGGRVWTNI----------PKGDIRRLNYPIQGTSAEGLKEALAIFYDKK---DPNWRIIAAIH 543

C_difficile 489 AEYIETLGGRYWSKETSEL------DKGSIKRYNYPIQGTSAEGLKEALYLLMKRK---PKDWLLVAIVH 549

C_perfringens 428 ADYIRSLGGRRWQGS----------NLTKTQRLNLPIQGSAAEGLKEALALLVERM---KDTWKLVAVVH 484

B_weidmannii 372 STNVFSKGGRTFNTYEC--------YMKASLKLNLPIQSSSAEGMKEALIYLYQNK---PKNWFICNCIH 430

T_pasteurii 334 DSHLLTPFGRIKP--LATF--------KPTQKRNYPFQSSIAVAVKQLMLIANK-------RFDVVHVIH 386

L_newyorkensis 318 EETLLTPFGLVKP--LAKF--------TQHQKRNFSFQAGVAVAIKKLMICLHP-------HFEIIHVIH 370

S_aureus_V 265 YKTMPTPYGIEHDMS----------AFQGSSIMAIYVQTVASYIFKHILLEVYKAQCE-KKTFKIIVPIH 323

S_aureus_mer 262 SNSMPMPYSIEHDMS----------AFQGSSIMAIYVQTVASYIFKHILLKVYKAQCD-QKTFKIIVPIH 320

[Consensus_aa:](http://prodata.swmed.edu/promals3d/info/consensus.html) .p.*l*.o..**G**..*h*s.............b.....*h***N***h*s*h***Q**tstt..*h***K**c*hhhhhh*..b.....p*@*.*llh*s*l***H**

[Consensus_ss:](http://prodata.swmed.edu/promals3d/info/consensus_ss.html) ee ee hhhhhhhhhhhhhhhhhhhhhhhhhh eeeeee

Active site: *****

1qsl 878 DELVFEVHKDDVDAVAKQIHQLMENCT---RLDVPL--LVEVGSGENWDQAH- 924

T_sanguinis 543 DELICEVPEEDAEYAKSVIEKIMINGMRKLVKQVPI--EVESHVSKVWT-K-- 590

S_qingdaonense 551 DEIILEVPDTNIEEASVFLNNVMVEGMKTLVKQIPI--EVELQSAKYWC-K-- 598

M_jejuensis 549 DEIVLEVPLADANQAKEILLETMQRGMSQIIPSIPI--EVDIKIESYWS-K-- 596

P_pinistramenti543 DELVLEVPEEKAEAAATFLANVMKEGMSRLIDKVPI--QVDVRINPYWN-KG- 591

N_nealsonii 544 DEIVVEVPKEDVLVAKDRLIKAMKLGMSYFITDVPI--EVDIDISEFWK-K-- 591

C_difficile 550 DEIVLEVPCKDEDVAKEILRDCMVEGMSKMVKKVPI--EVDVKSSITWD-KP- 598

C_perfringens 485 DEILLEVPEDESEEAKNILESCMIEGMQIIVKDIPI--VVDSTISNNWC-K-- 532

B_weidmannii 431 DQIYLEVPNKDIEEAKELLEQSMILGMEKFIKSVPV--SVTLEVIKN------ 475

T_pasteurii 387 DEIWILAPTRE-PTVVEEVIEEFYHQLLNILPDFPISGFIKVEPIGGNTHEHG 438

L_newyorkensis 371 DELWVLAYPED-RVLLDTMIAQFYEEMTVILPKFPTKDFVKIELLGGV----- 417

S_aureus_V 324 DAIMIEC---NDKGIAQNVAQLMKDTANQLFNGEFA--HVTVEALGGI-DNE- 369

S_aureus_mer 321 DAIMIEC---EDEEIAQNVGQLMKDTANQLFNGEFA--HVTVEEIGGV-DHE- 366

[Consensus_aa:](http://prodata.swmed.edu/promals3d/info/consensus.html) **D**p*lhl***E***h*..cc.p.*h*.p.*l*.p.**M**bps*h*p.*hh*.p*h***P***h*...**V**p*l*p....*h*..p..

[Consensus_ss:](http://prodata.swmed.edu/promals3d/info/consensus_ss.html) eeeee hhhhhhhhhhhhhhhhhhhhhh e eeeeee

**References:**

PROMALS3D: a tool for multiple sequence and structure alignment. Jimin Pei, Bong-Hyun Kim and Nick V. Grishin. Nucleic Acids Res. 2008 36(7):2295-2300.

PDB ID 1qsl: [Structural elucidation of the binding and inhibitory properties of lanthanide (III) ions at the 3'-5' exonucleolytic active site of the Klenow fragment.](https://pubmed.ncbi.nlm.nih.gov/10631518/)

Brautigam CA, Aschheim K, Steitz TA.Chem Biol. 1999 Dec;6(12):901-8. doi: 10.1016/s1074-5521(00)80009-5.PMID: 10631518
