# Supplementary material for: Mobile genetic element-encoded putative DNA primases composed of A-family polymerase—SSB pairs
Source: Front Mol Biosci. 2023 Mar 16;10:1113960. doi: 10.3389/fmolb.2023.1113960 (PMC10061031; doi:10.3389/fmolb.2023.1113960)
Supplement: Supplementary file 2 [file DataSheet3.PDF]

Figure S3. (legend on p4)

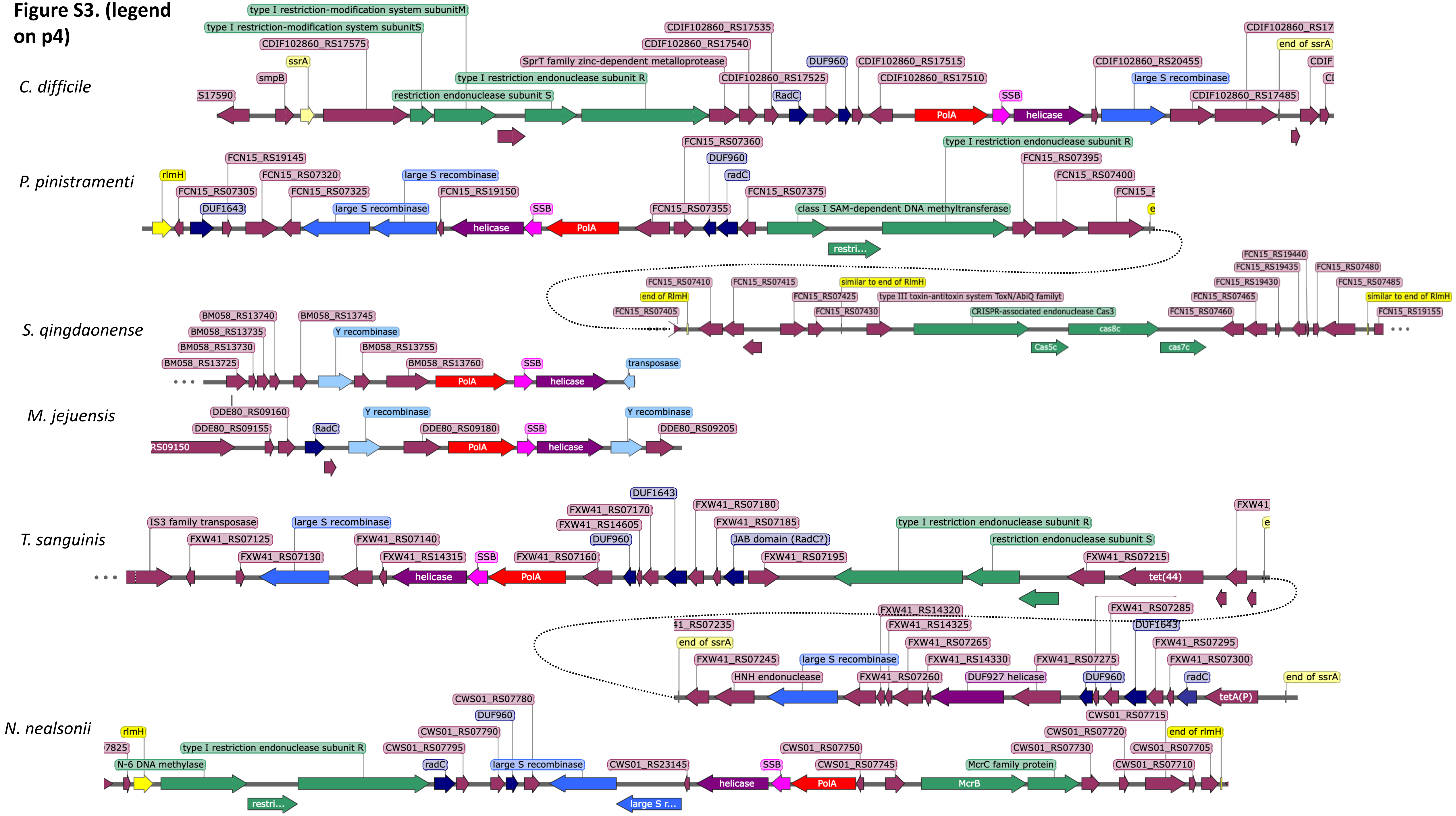

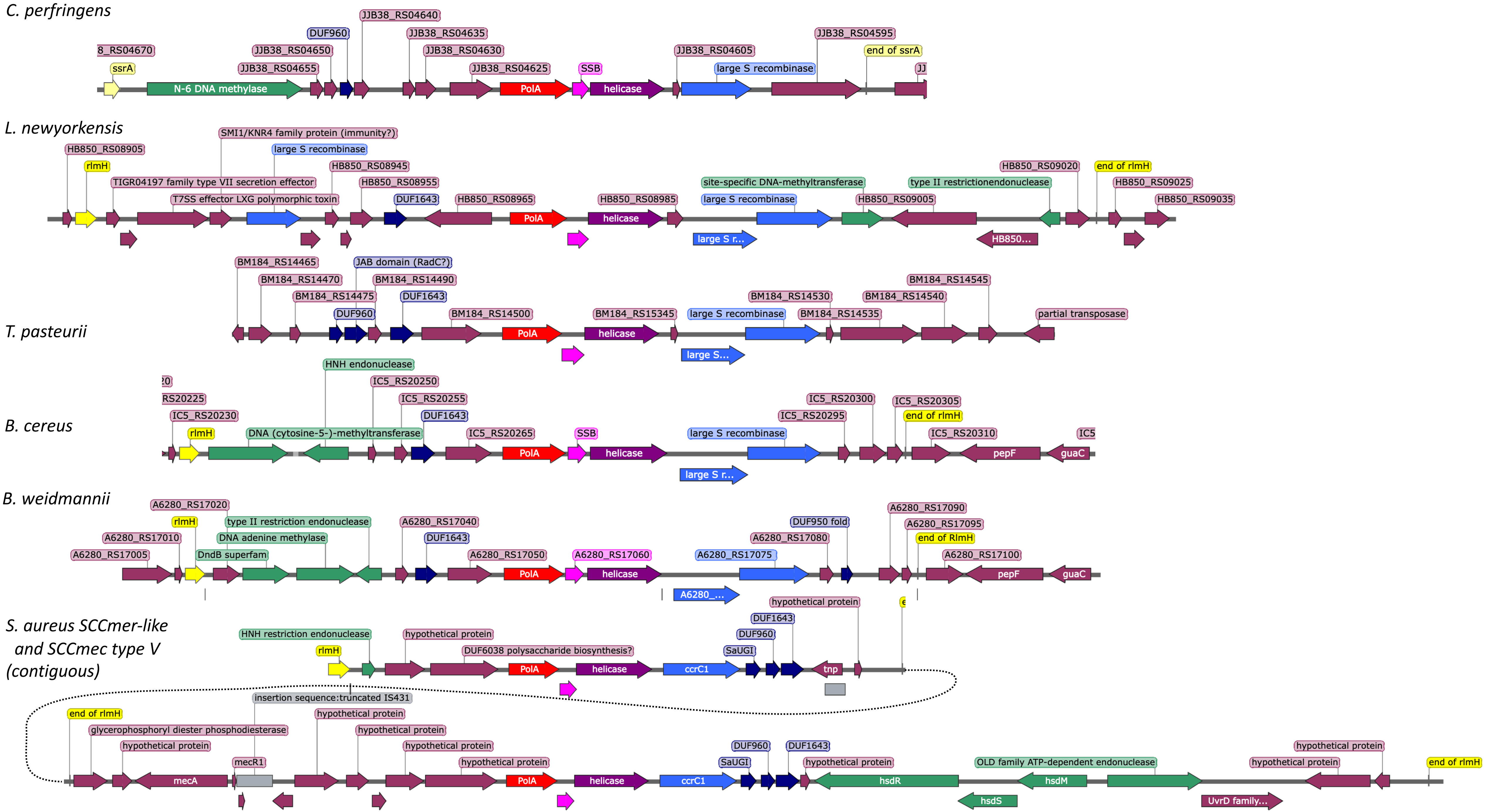
C. perfringens
8_RS04670
ssrA
N-6 DNA methylase
JJB38_RS04655
JJB38_RS04650
DUF960
JJB38_RS04640
JJB38_RS04635
JJB38_RS04630
JJB38_RS04625
PolA
SSB
helicase
JJB38_RS04605
large S recombinase
JJB38_RS04595
end of ssrA
JJ
L. newyorkensis
HB850_RS08905
rlmH
TIGR04197 family type VII secretion effector
SMI1/KNR4 family protein (immunity?)
T7SS effector LXG polymorphic toxin
large S recombinase
HB850_RS08945
HB850_RS08955
DUF1643
HB850_RS08965
PolA
HB850_RS08985
helicase
site-specific DNA-methyltransferase
large S recombinase
large S r...
HB850_RS09005
type II restrictionendonuclease
HB850...
HB850_RS09020
end of rlmH
HB850_RS09025
HB850_RS09035
T. pasteurii
BM184_RS14465
BM184_RS14470
BM184_RS14475
DUF960
JAB domain (RadC?)
BM184_RS14490
DUF1643
BM184_RS14500
PolA
BM184_RS15345
helicase
large S recombinase
large S...
BM184_RS14530
BM184_RS14535
BM184_RS14540
BM184_RS14545
partial transposase
B. cereus
20
RS20225
IC5_RS20230
rlmH
DNA (cytosine-5-)-methyltransferase
HNH endonuclease
IC5_RS20250
IC5_RS20255
DUF1643
IC5_RS20265
PolA
SSB
helicase
large S recombinase
large S r...
IC5_RS20295
IC5_RS20300
IC5_RS20305
end of rlmH
IC5_RS20310
pepF
IC5
guaC
B. weidmannii
A6280_RS17005
A6280_RS17010
A6280_RS17020
rlmH
DndB superfam
DNA adenine methylase
type II restriction endonuclease
A6280_RS17040
DUF1643
A6280_RS17050
PolA
A6280_RS17060
helicase
A6280_RS17075
A6280_...
A6280_RS17080
DUF950 fold
A6280_RS17090
A6280_RS17095
end of RlmH
A6280_RS17100
pepF
guaC
S. aureus SCCmer-like
and SCCmec type V
(contiguous)
rlmH
HNH restriction endonuclease
hypothetical protein
DUF6038 polysaccharide biosynthesis?
PolA
helicase
ccrC1
SaUGI
DUF960
DUF1643
hypothetical protein
tnp
e
insertion sequence:truncated IS431
end of rlmH
glycerophosphoryl diester phosphodiesterase
hypothetical protein
mecA
mecR1
hypothetical protein
hypothetical protein
hypothetical protein
hypothetical protein
PolA
helicase
ccrC1
SaUGI
DUF960
DUF1643
hypothetical protein
hsdR
OLD family ATP-dependent endonuclease
hsdM
hsdS
UvrD family...
hypothetical protein
hypothetical protein
end of rlmH

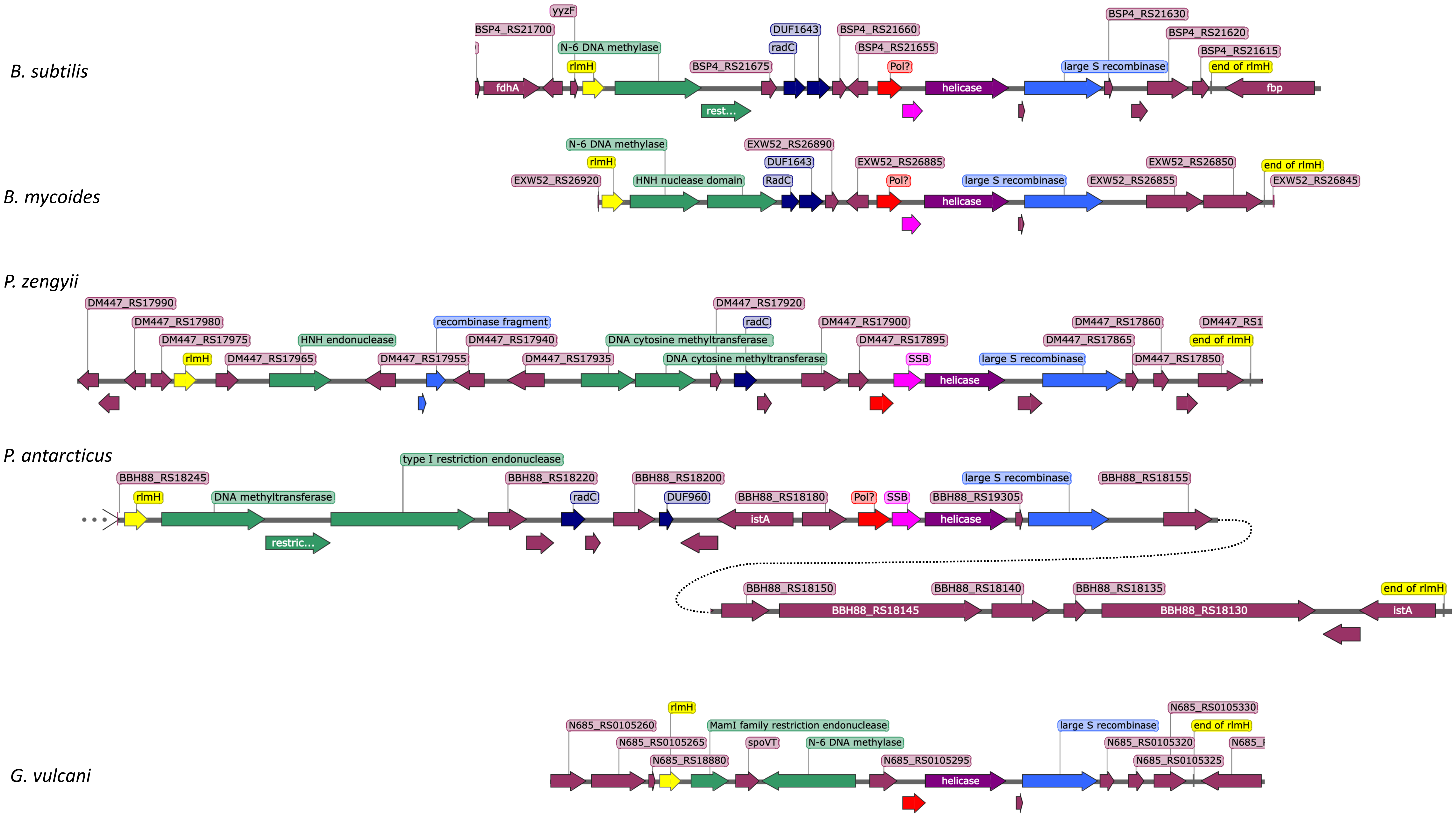
B. subtilis
yyzF
BSP4_RS21700
N-6 DNA methylase
rlmH
fdhA
rest...
BSP4_RS21675
DUF1643
radC
BSP4_RS21660
BSP4_RS21655
Pol?
helicase
BSP4_RS21630
large S recombinase
BSP4_RS21620
BSP4_RS21615
end of rlmH
fbp
B. mycoides
N-6 DNA methylase
rlmH
EXW52_RS26920
HNH nuclease domain
EXW52_RS26890
DUF1643
RadC
EXW52_RS26885
Pol?
helicase
large S recombinase
EXW52_RS26855
EXW52_RS26850
end of rlmH
EXW52_RS26845
P. zengyii
DM447_RS17990
DM447_RS17980
DM447_RS17975
rlmH
DM447_RS17965
HNH endonuclease
recombinase fragment
DM447_RS17955
DM447_RS17940
DM447_RS17935
DM447_RS17920
radC
DNA cytosine methyltransferase
DNA cytosine methyltransferase
DM447_RS17900
DM447_RS17895
SSB
helicase
large S recombinase
DM447_RS17860
DM447_RS17865
DM447_RS17850
DM447_RS1
end of rlmH
P. antarcticus
BBH88_RS18245
rlmH
DNA methyltransferase
restric...
type I restriction endonuclease
BBH88_RS18220
radC
BBH88_RS18200
DUF960
BBH88_RS18180
istA
Pol?
SSB
BBH88_RS19305
helicase
large S recombinase
BBH88_RS18155
BBH88_RS18150
BBH88_RS18145
BBH88_RS18140
BBH88_RS18135
BBH88_RS18130
end of rlmH
istA
G. vulcani
rlmH
N685_RS0105260
N685_RS0105265
N685_RS18880
MamI family restriction endonuclease
spoVT
N-6 DNA methylase
N685_RS0105295
helicase
large S recombinase
N685_RS0105330
end of rlmH
N685_RS0105320
N685_RS0105325
N685_

Figure S3. Genomic context of Pol-SSB pairs. Diagrams of the region encoding each pair are shown, colored to highlight relevant features (if found). Red, PolA (or minimal Pol-like protein); pink, SSB; purple, helicase; bright blue, large serine recombinase; yellow, putative insertion sites, pale blue, other recombinase possibly involved in mobility; dark blue, homology to genes found in the recombinase locus of SCC elements. For extremely long elements, dotted lines simply connect one segment to the next (they do not signify gaps). See table 1 for accession numbers.
