# Supplementary material for: Mobile genetic element-encoded putative DNA primases composed of A-family polymerase—SSB pairs
Source: Front Mol Biosci. 2023 Mar 16;10:1113960. doi: 10.3389/fmolb.2023.1113960 (PMC10061031; doi:10.3389/fmolb.2023.1113960)
Supplement: Supplementary file 3 [file DataSheet2.DOCX]

Figure S2: Predicted Alignment Error plots for all predicted complex structures.

In all cases, protein A is the putative polymerase and B is the putative SSB.

Plots are shown for all 5 models predicted for each complex.

*Please see the last page for a guide to interpreting these plots.*

*S. aureus* SCC*mec* type V


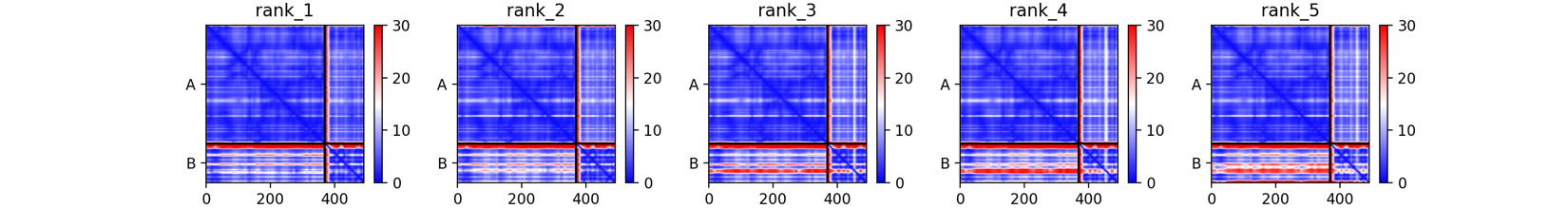


*S. aureus* SCC*mer* type V


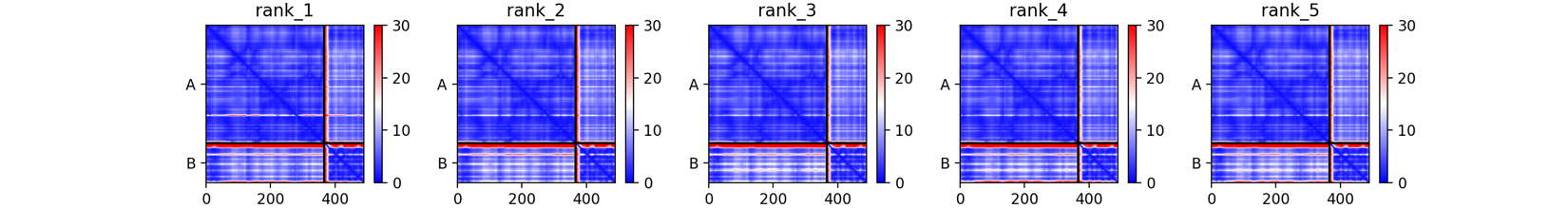


*Listeria newyorkensis* strain FSL L7-1614
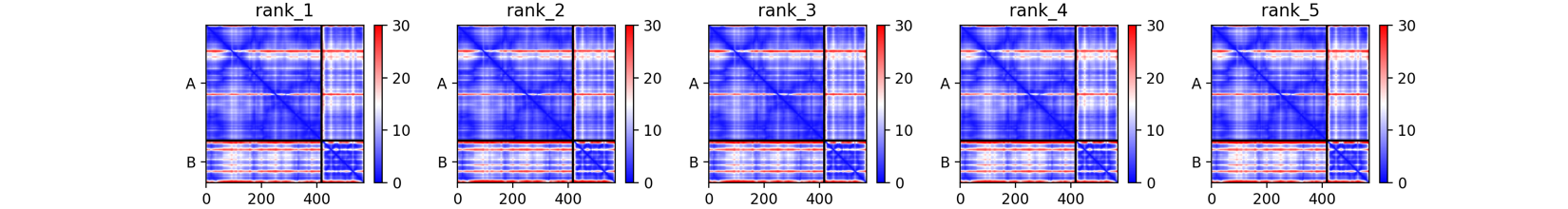


*Trichococcus pasteurii* strain DSM 2381


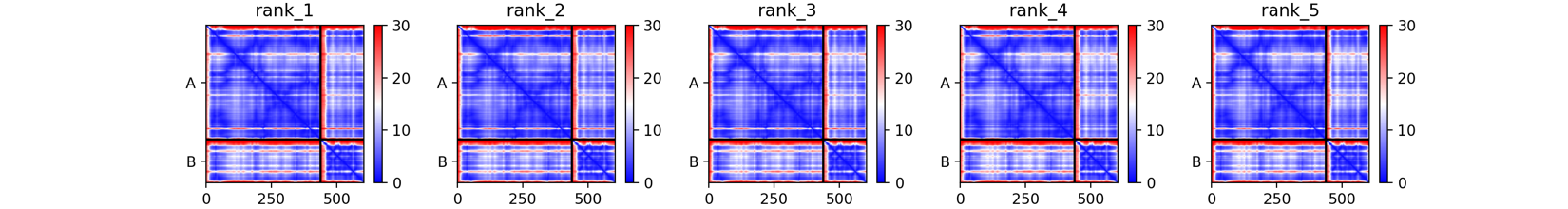


*Bacillus Weidmannii* strain FSL J3-0113


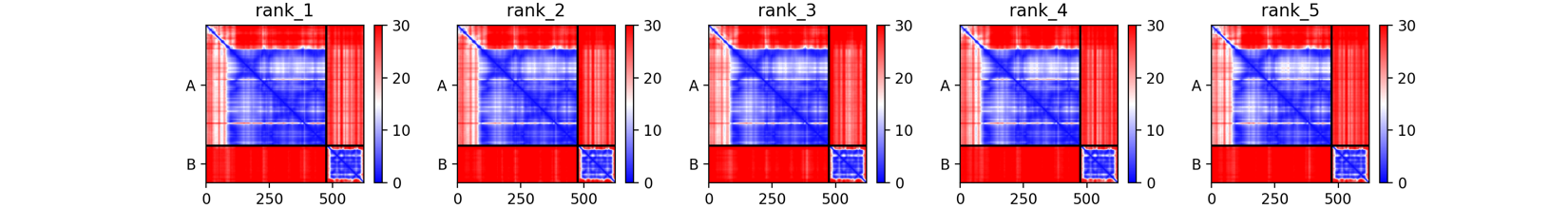


*Clostridium perfringens* strain CHD32500R


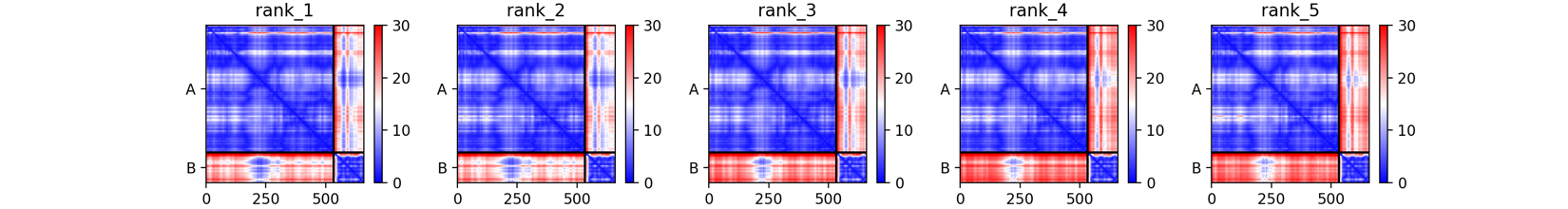


*Niallia nealsonii*


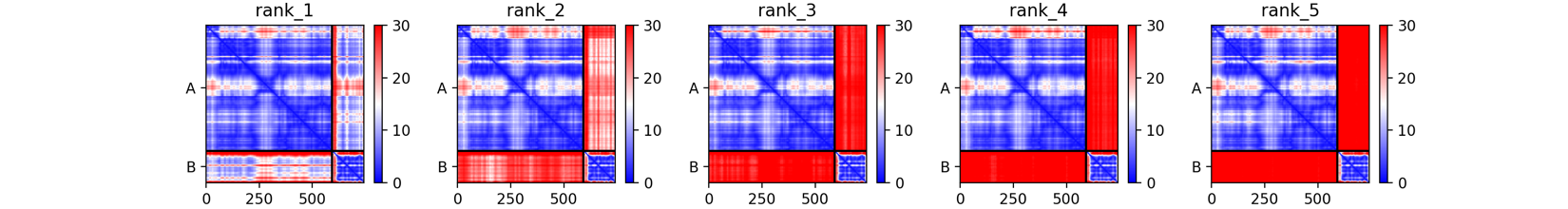


*Clostridiodes difficile* DSM102860


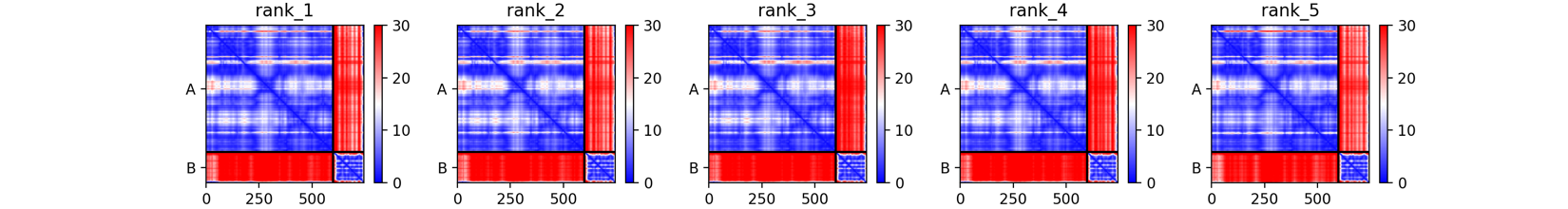


*Paenibacillus pinistramenti*


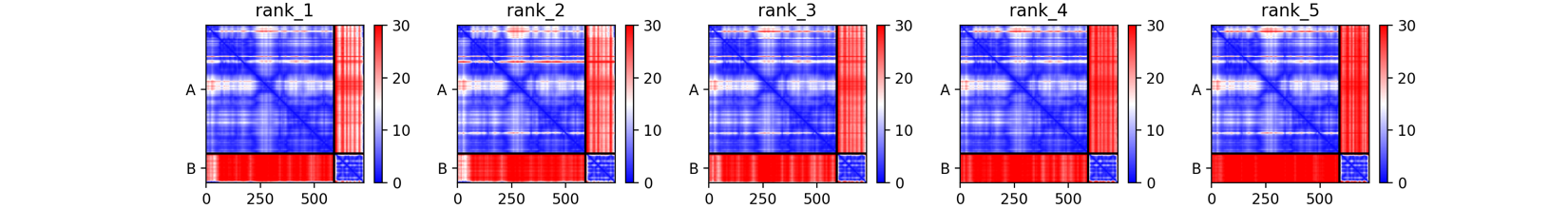


*Salibacterium qingdaonense* strain CGMCC 1.6134


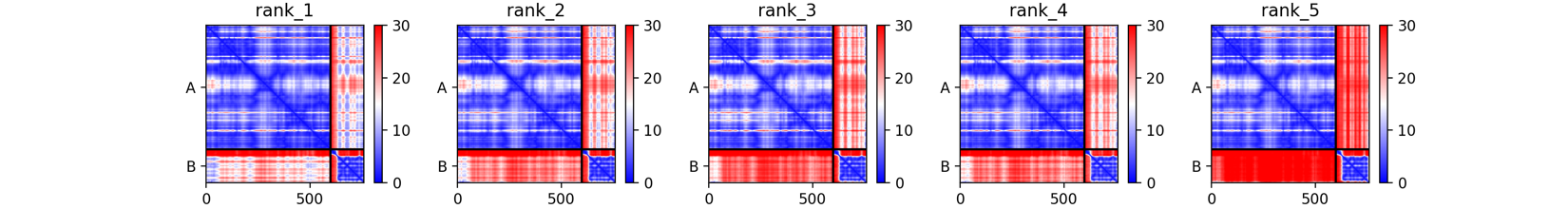


*Metalysinibacillus jejuensis* strain N25


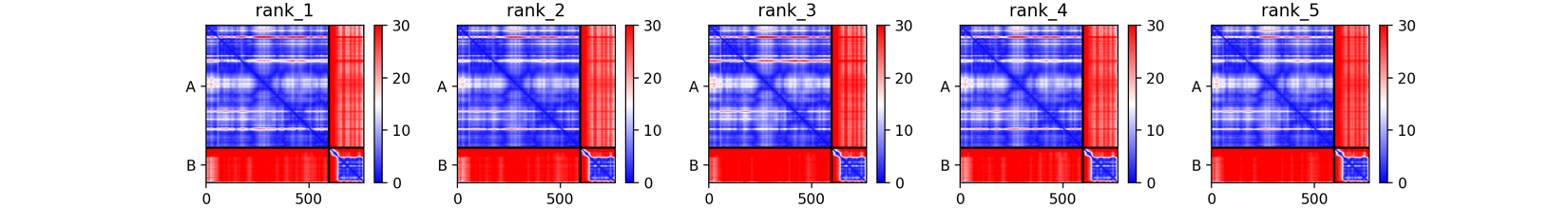


*Turicibacter sanguinis* isolate MGYG-HGUT-00143


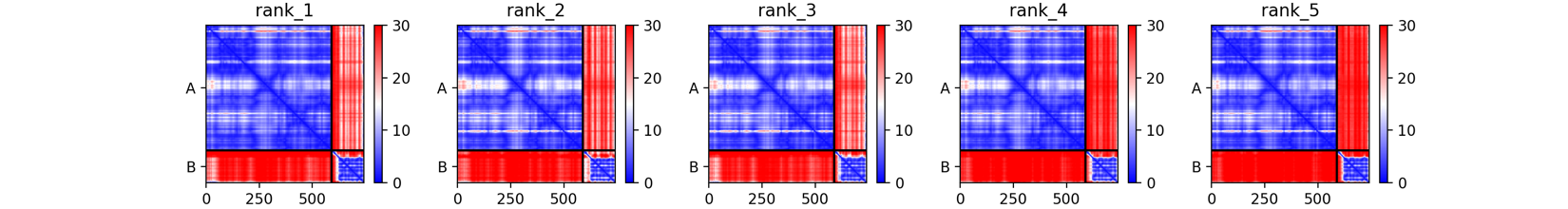


**Putative Pol proteins with little beyond the RRM motif (no protein-protein contacts predicted for any of the following)**

*Bacillus subtilis* strain Bsp4


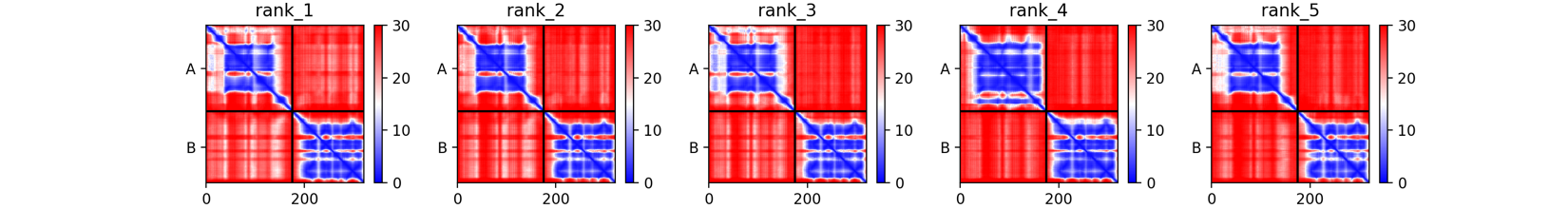


*Bacillus mycoides* strain BPN51/1


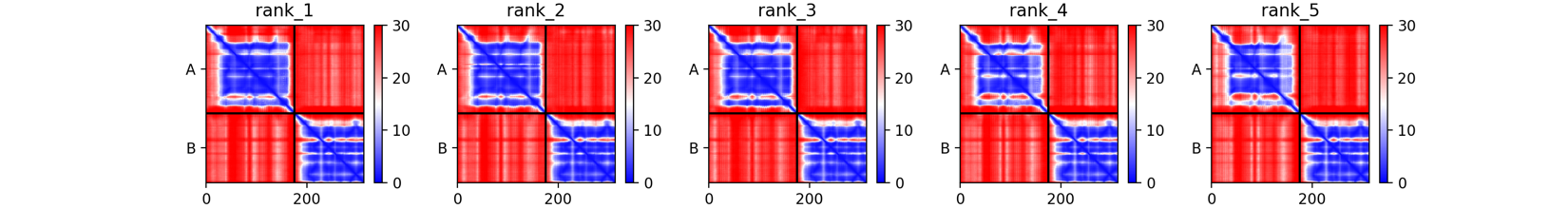


*Paraliobacillus zengyii* strain X-1125


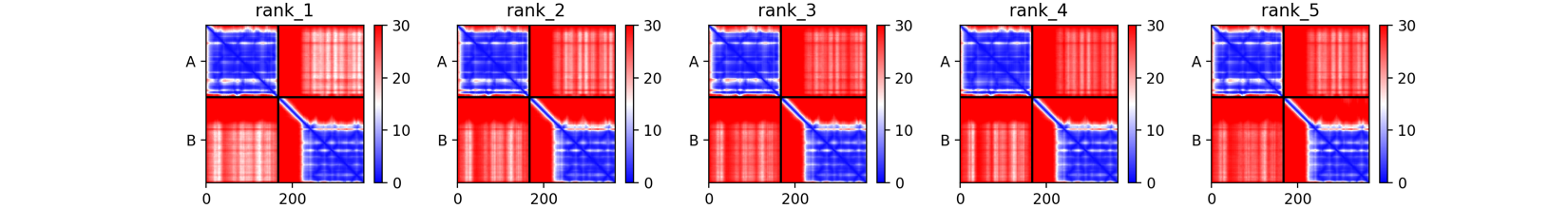


*Planococcus antarcticus* DSM 14505


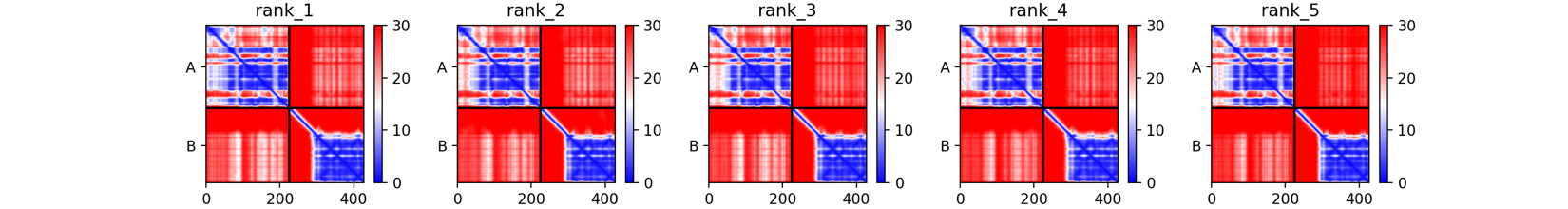


*Geobacillus vulcani* PSS1 (no SSB)


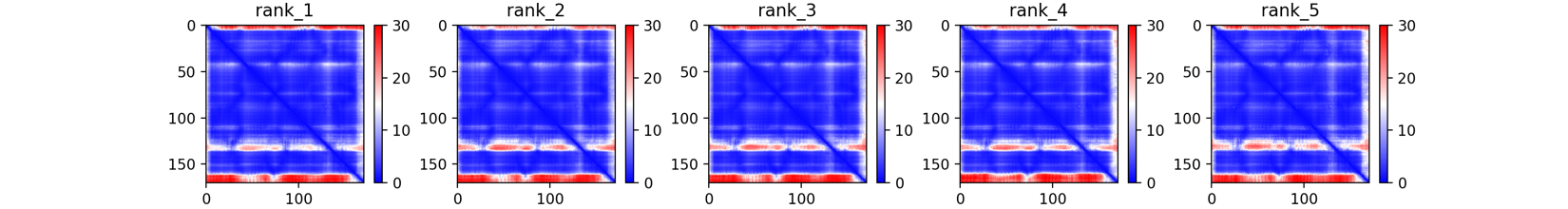


**Guide to interpreting PAE (predicted alignment error plots) from AlphaFold**, using the top model for the *C. difficile* complex as an example. The predicted error in relative positioning between all pairs of residues is color-coded from blue (low) to red (high). On-diagonal boxes reflect the folding of individual proteins (or domains) and off-diagonal boxes reflect the positioning of one protein (or domain) relative to the others. Protein A is the predicted polymerase and B is the predicted SSB. Green boxes highlight the various domains or subdomains of the polymerase. Yellow off-diagonal boxes highlight less-blue sections of the plot which indicate that the coiled coil portion of the thumb is predicted to be connected to the rest of the polymerase by a flexible hinge.

The small off-diagonal green boxes highlight the predicted interaction between the C-terminal extension of the SSB with the N-terminal extension of the polymerase.


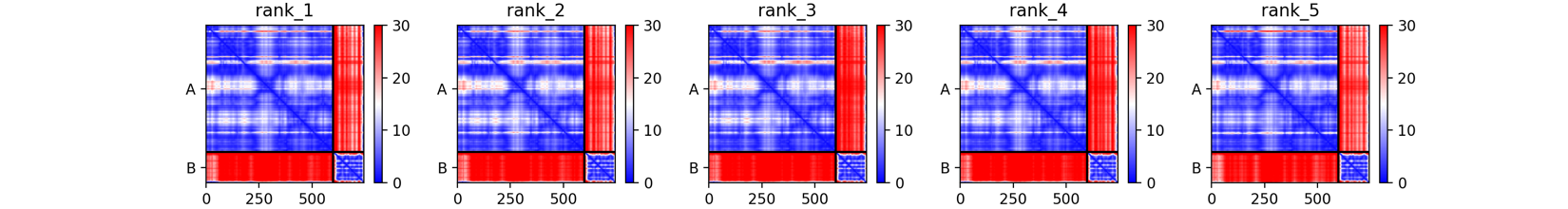


N-ter

N-ter

exo

exo

Thumb

Coiled coil

Thumb

Thumb

Beta hairpin

Fingers & palm

Fingers & palm

SSB

SSB

**C-ter of SSB binds N-ter of Pol**


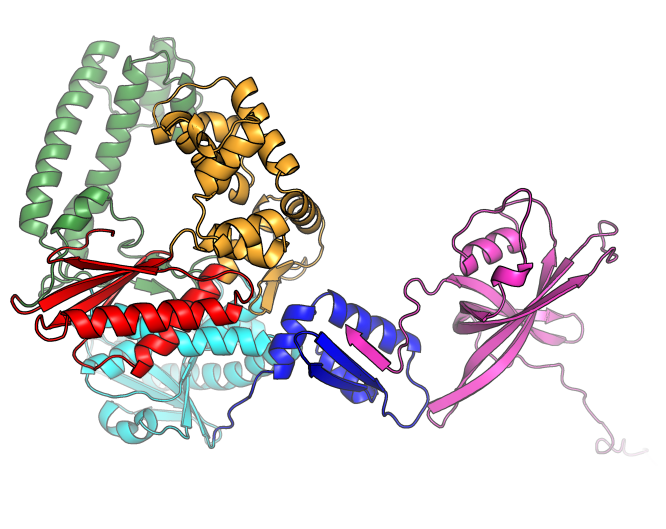


N

C

N

C
