# Supplementary material for: Mobile genetic element-encoded putative DNA primases composed of A-family polymerase—SSB pairs
Source: Front Mol Biosci. 2023 Mar 16;10:1113960. doi: 10.3389/fmolb.2023.1113960 (PMC10061031; doi:10.3389/fmolb.2023.1113960)
Supplement: Supplementary file 4 [file DataSheet1.DOCX]

Figure S1. Pairwise sequence identities among proteins studied, as calculated by Clustal Omega

Pol proteins containing intact polymerase domains (after removal of the variable N-termini)

1: Staphylococcus_aureus_V 100.00 79.03 18.06 17.33 16.44 18.67 16.72 16.83 17.16 18.48 16.89 16.50

2: Staphylococcus_aureus_mer 79.03 100.00 19.40 18.00 16.11 18.67 17.39 15.84 18.15 19.14 17.55 15.51

3: Listeria_newyorkensis 18.06 19.40 100.00 40.05 20.11 18.48 20.05 16.94 16.09 18.77 20.43 21.51

4: Trichococcus_pasteurii 17.33 18.00 40.05 100.00 23.16 22.62 23.37 20.97 18.87 22.37 22.37 20.43

5: Bacillus_wiedmannii 16.44 16.11 20.11 23.16 100.00 34.13 38.30 36.41 35.09 35.88 36.17 32.19

6: Turicibacter_sanguinis 18.67 18.67 18.48 22.62 34.13 100.00 40.80 42.89 40.53 42.11 39.36 41.05

7: Clostridium_perfringens 16.72 17.39 20.05 23.37 38.30 40.80 100.00 50.13 43.01 42.48 43.24 43.01

8: Clostridioides_difficile 16.83 15.84 16.94 20.97 36.41 42.89 50.13 100.00 51.56 51.56 51.58 48.57

9: Salibacterium_qingdaonense 17.16 18.15 16.09 18.87 35.09 40.53 43.01 51.56 100.00 55.06 44.74 51.04

10: Metalysinibacillus_jejuensis 18.48 19.14 18.77 22.37 35.88 42.11 42.48 51.56 55.06 100.00 50.79 52.60

11: Niallia_nealsonii 16.89 17.55 20.43 22.37 36.17 39.36 43.24 51.58 44.74 50.79 100.00 52.63

12: Paenibacillus_pinistramenti 16.50 15.51 21.51 20.43 32.19 41.05 43.01 48.57 51.04 52.60 52.63 100.00

Putative, possibly vestigial Pol proteins containing little beyond an RRM

1: Bacillus_subtilis 100.00 53.71 13.38 14.77 9.87

2: Bacillus_mycoides 53.71 100.00 13.38 14.77 12.50

3: Planococcus_antarcticus 13.38 13.38 100.00 21.33 32.45

4: Paraliobacillus_zengyii 14.77 14.77 21.33 100.00 28.74

5: Geobacillus_vulcani 9.87 12.50 32.45 28.74 100.00

SSB proteins

1: Listeria_newyorkensis 100.00 32.43 9.20 9.09 10.74 10.53 16.53 16.39 15.70 16.42 16.24 12.80 16.54 16.28 15.50 13.18

2: Trichococcus_pasteurii 32.43 100.00 12.77 13.68 9.60 11.86 13.74 15.79 15.15 15.27 14.66 12.90 14.17 13.18 12.32 14.71

3: Staphylococcus_aureus_V 9.20 12.77 100.00 68.85 13.41 9.76 13.83 12.37 13.68 9.09 10.23 20.22 13.83 12.77 14.00 16.49

4: Staphylococcus_aureus_mer 9.09 13.68 68.85 100.00 12.05 10.84 8.42 13.27 12.50 6.74 7.87 13.33 16.84 14.74 15.84 16.33

5: Bacillus_subtilis 10.74 9.60 13.41 12.05 100.00 69.57 15.75 14.49 14.71 14.84 24.58 20.80 11.72 15.50 15.15 16.03

6: Bacillus_mycoides 10.53 11.86 9.76 10.84 69.57 100.00 15.57 16.79 16.91 12.40 21.19 21.01 14.75 15.57 12.80 16.13

7: Turicibacter_sanguinis 16.53 13.74 13.83 8.42 15.75 15.57 100.00 19.58 21.99 16.15 16.81 15.20 16.03 20.45 18.44 18.44

8: Paraliobacillus_zengyii 16.39 15.79 12.37 13.27 14.49 16.79 19.58 100.00 36.41 16.79 17.60 20.31 16.90 18.05 16.56 17.79

9: Planococcus_antarcticus 15.70 15.15 13.68 12.50 14.71 16.91 21.99 36.41 100.00 17.97 23.58 19.05 20.14 16.92 16.89 20.00

10: Bacillus_wiedmannii 16.42 15.27 9.09 6.74 14.84 12.40 16.15 16.79 17.97 100.00 21.95 24.06 21.48 19.57 17.65 14.60

11: Clostridium_perfringens 16.24 14.66 10.23 7.87 24.58 21.19 16.81 17.60 23.58 21.95 100.00 23.58 23.02 23.81 25.60 20.00

12: Paenibacillus_pinistramenti 12.80 12.90 20.22 13.33 20.80 21.01 15.20 20.31 19.05 24.06 23.58 100.00 34.56 31.62 20.00 24.81

13: Niallia_nealsonii 16.54 14.17 13.83 16.84 11.72 14.75 16.03 16.90 20.14 21.48 23.02 34.56 100.00 34.04 24.83 25.17

14: Clostridioides_difficile 16.28 13.18 12.77 14.74 15.50 15.57 20.45 18.05 16.92 19.57 23.81 31.62 34.04 100.00 21.83 24.29

15: Salibacterium_qingdaonense 15.50 12.32 14.00 15.84 15.15 12.80 18.44 16.56 16.89 17.65 25.60 20.00 24.83 21.83 100.00 30.13

16: Metalysinibacillus_jejuensis 13.18 14.71 16.49 16.33 16.03 16.13 18.44 17.79 20.00 14.60 20.00 24.81 25.17 24.29 30.13 100.00
